# Comparison of the clinical efficacy and safety of two spinal endoscopic techniques for the treatment of ossification of the ligamentum flavum in the thoracic spine

**DOI:** 10.3389/fneur.2025.1630315

**Published:** 2025-07-23

**Authors:** Yupeng Wang, Zhongxin Tang, Qian Tang, Haijun Ma, Mingkui Shen, Hejun Yang

**Affiliations:** Department of Mini-Invasive Spinal Surgery, The Third People's Hospital of Henan Province, Zhengzhou, China

**Keywords:** thoracic ossification of the ligamentum flavum, unilateral biportal endoscopic, percutaneous endoscopic, clinical efficacy, minimally invasive spine surgery

## Abstract

**Purpose:**

The purpose of this study was to compare the clinical efficacy and safety of the percutaneous endoscopic (PE) technique and the unilateral biportal endoscopic (UBE) technique for the treatment of thoracic ossification of the ligamentum flavum (T-OLF).

**Methods:**

This study retrospectively analyzed T-OLF patients who received endoscopic surgical treatment from January 1, 2020, to December 31, 2022. Patients were divided into a PE group and a UBE group according to the surgical method. The basic information of the patients was recorded. Preoperative and postoperative neurological status was evaluated using the mJOA score, American Spinal Injury Association (ASIA) sense score (ASS), and ASIA motor score of the lower extremities (AMS). The mJOA recovery rate (RR) was also calculated. T-OLF can be diagnosed on the basis of sagittal Magnetic Resonance Imaging (MRI) and axial Computed Tomography (CT).

**Results:**

A total of 64 patients were followed for an average of 12–24 months. 33 patients underwent UBE surgery, and 31 patients underwent PE surgery. There was no significant difference in baseline characteristics between the two groups (*p* > 0.05). Neurological function was significantly improved in both groups after surgery. Compared with the PE group, the UBE group experienced better postoperative clinical efficacy, and the difference was statistically significant (*p* < 0.05). Postoperative CT and MRI revealed that the degree of spinal cord compression in patients in the UBE and PE groups was relieved. 4 patients in the PE group and 1 patient in the UBE group had dural sac tears. 3 patients with upper thoracic vertebrae in the PE group exhibited transient neurological deficits. These complications did not cause serious consequences.

**Conclusion:**

For patients with T-OLF, both UBE and PE can effectively alleviate nerve compression and relieve symptoms. UBE uses two channels for observation and operation, leading to more effective and safer clinical outcomes.

## Introduction

Thoracic ossification of the ligamentum flavum (T-OLF), which leads to thoracic spinal canal stenosis, was first reported by Le Double in 1912 (1). T-OLF is a relatively rare spinal disease worldwide that is most commonly found in East Asian countries and typically affects the lower thoracic spine (2, 3). The average age of onset is between 50 and 60 years (4). The rarity and insidious onset of T-OLF often lead to delayed diagnosis. Therefore, by the time clinical symptoms present, most patients need surgical intervention to relieve spinal cord compression (5). Previous studies have demonstrated that the duration of preoperative symptoms, the severity of spinal cord compression, and changes in MRI signals in the spinal cord influence surgical outcomes (6, 7). Consequently, early and thorough decompression is essential for effective treatment. Posterior laminectomy is widely regarded as the standard treatment for T-OLF (8). However, excessive disruption of the muscle–ligament complex and facet joints during this procedure can lead to complications, including thoracic back pain and local kyphotic deformity, which may adversely affect patient outcomes (9). Although fusion surgery can prevent local kyphotic deformities, it does not alleviate thoracic back pain and may, in fact, contribute to adjacent segment disease (10).

With the development of minimally invasive technology, endoscopic surgery for T-OLF has demonstrated promising outcomes (11, 12). Percutaneous endoscopic (PE) surgery for T-OLF has been documented in multiple studies (13, 14). Endoscopic instruments can minimize damage to paravertebral muscles and bone structures, thereby advancing the application of endoscopic technology in the treatment of thoracic spine diseases. Unilateral biportal endoscopic (UBE) decompression is a novel technique performed using a percutaneous endoscope (15). Unlike PE uniaxial endoscopic approaches, it offers a greater operating range, enhanced flexibility, and a wider, clearer surgical field and has been extensively utilized in the treatment of lumbar spinal stenosis (16).

However, few reports exist on the use of UBE for thoracic spinal canal decompression, and few studies are available that compare the effectiveness and safety of these two endoscopic techniques for the treatment of T-OLF. Therefore, the purpose of this study was to compare the clinical efficacy and safety of two spinal endoscopic techniques for the treatment of T-OLF.

## Methods

### Patients

A total of 64 patients with T-OLF who underwent thoracic spine surgery at our institution from January 1, 2020, to December 31, 2023, were analyzed. The patients were divided into two groups according to the surgical method used: the UBE group (*n* = 33) and the PE group (*n* = 31). All patients met the clinical and radiological standards for T-OLF and underwent surgery performed by an experienced spinal team. This study was approved by the Medical Ethics Committee of the Third People’s Hospital of Henan Province, and all the patients provided informed consent. The minimum follow-up time was 1 year.

The inclusion criteria were as follows: (1) CT reveals ossification of the ligamentum flavum in a segment of the thoracic spine, and MRI reveals compression of the thoracic spinal cord; (2) unilateral or bilateral sensory and motor disorders of the body and lower limbs, with hyperreflexia of the lower limbs and positive pathological signs; and (3) physical signs match the imaging symptoms.

The exclusion criteria were as follows: (1) patients with tumors, severe organic diseases, severe cardiovascular or cerebrovascular diseases, hemorrhagic diseases, etc.; (2) patients with spinal instability or deformity; (3) patients with thoracic ossification of the posterior longitudinal ligament, thoracic disc herniation or other thoracic diseases caused by ventral spinal cord compression; and (4) patients with incomplete follow-up data.

### Surgical methods

#### UBE

**Figure 1:**
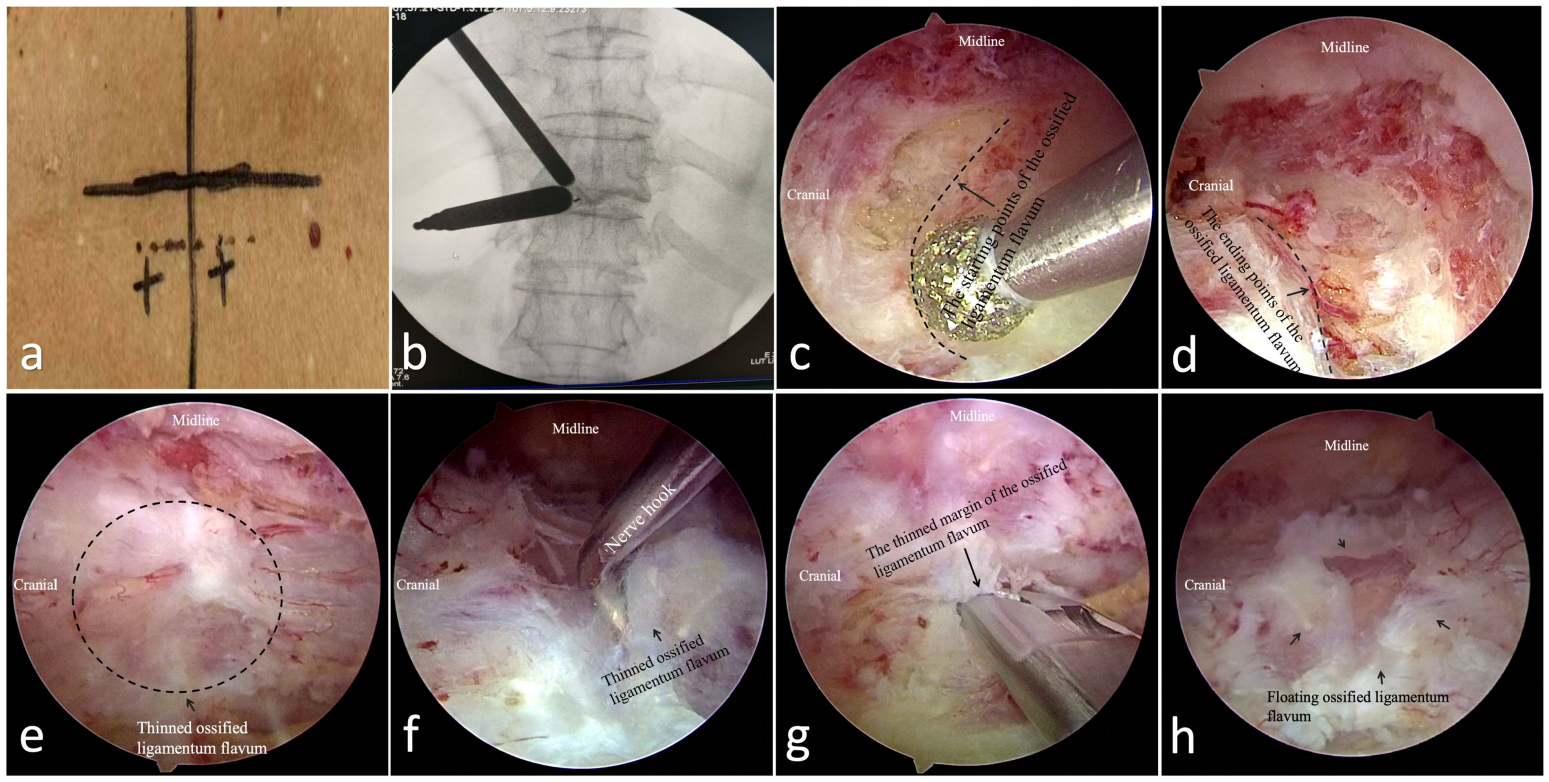
**(a)** Lineation-on-body; **(b)** Cannulas converging on the surface of the lamina; **(c)** The lamina at the head; **(d)** The lamina at the end; **(e)** The thinned ossified ligamentum flavum; **(f)** Use a nerve hook to separate the deep surface of the ossified ligamentum flavum; **(g)** Remove the ossified ligamentum flavum with a Kerrison rongeur; **(h)** Complete decompression of the spinal cord.

**Figure 2:**
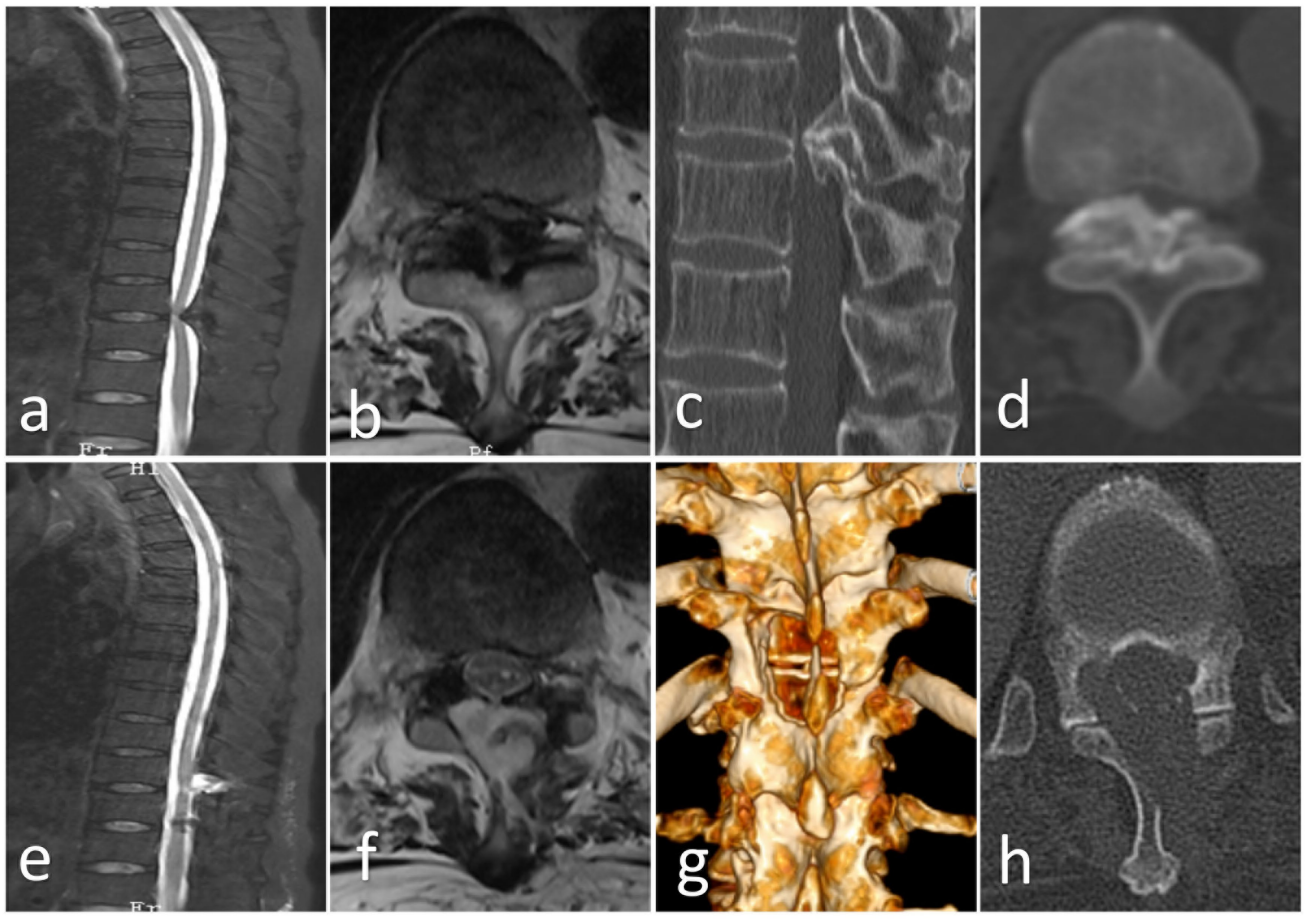
**(a,b)** Preoperative MRI shows ossification of the thoracic ligamentum flavum (Round); **(c,d)** Preoperative CT shows severe ossification of the thoracic ligamentum flavum (Fused); **(e,f)** Postoperative CT shows that the ossification has been removed and spinal cord decompression has been successful, and a small amount of cerebrospinal fluid leaked out; **(g,h)** Postoperative CT and 3D reconstruction show that the spinal canal has been opened and the ossification has been removed.

The operation was performed with conventional intubation under general anesthesia. The patient was placed in the prone position with a suitable body position pad. By adjusting the operating table, the intervertebral space of the target segment was made perpendicular to the ground as much as possible. Taking the position of ossification of ligamentum flavum as the horizontal line, a longitudinal incision was selected. The operation channel and observation channel are approximately 2.0 cm away from the midline of the spinous process, the distal end is used as the operation channel, the proximal end is used as the observation channel, and the distance between the midpoint of the two incisions is approximately 3.0 cm (Figure 1a). After routine disinfection and draping with a waterproof membrane, an operating channel (approximately 1.0 cm incision) and an observation channel (approximately 5.0 mm incision) are established, with the tips of the cannulas converging on the surface of the lamina (Figure 1b). After confirming the proper positioning of the cannulae under C-arm fluoroscopy, the visual field was located under the endoscope. A 90° plasma radiofrequency electrode is then used to expose the lamina and the base of the spinous process. Next, a grinding drill is used to create a depression. Anteroposterior and lateral fluoroscopy were performed to verify the correct segment and confirm the starting position. A 4.0 mm grinding drill was subsequently used to quickly remove the superficial lamina and cancellous bone. The lamina at the head (Figure 1c) and tail (Figure 1d) were removed to the starting and ending points of the ligamentum flavum, and the lateral boundary extended to the medial edge of the facet joint. The grinding range of the base of the spinous process should ensure that the access of instruments is not blocked. Next, a grinding drill was used to adequately thin the ossified ligamentum flavum (Figure 1e). Finally, a nerve hook was used to separate the deep surface of the ossified ligamentum flavum (Figure 1f), and the ossified ligamentum flavum was gradually removed with a Kerrison rongeur. If the ossified ligamentum flavum strongly adheres to the dura mater or if there is ossification of the dura mater, it can be adequately thinned using a grinding drill and circumferentially dissected for floating treatment (Figure 1g). If there is hypertrophic adhesion of the bilateral facet joints, they can first be thinned with a grinding drill, and then decompression of the nerve root canal can be performed using a Kerrison rongeur. Finally, complete decompression of the spinal canal was ensured (Figure 1h), hemostasis was obtained, a drain was placed, the endoscope was removed, the wound was sutured, and the operation was complete (Figure 2).

#### PE

**Figure 3:**
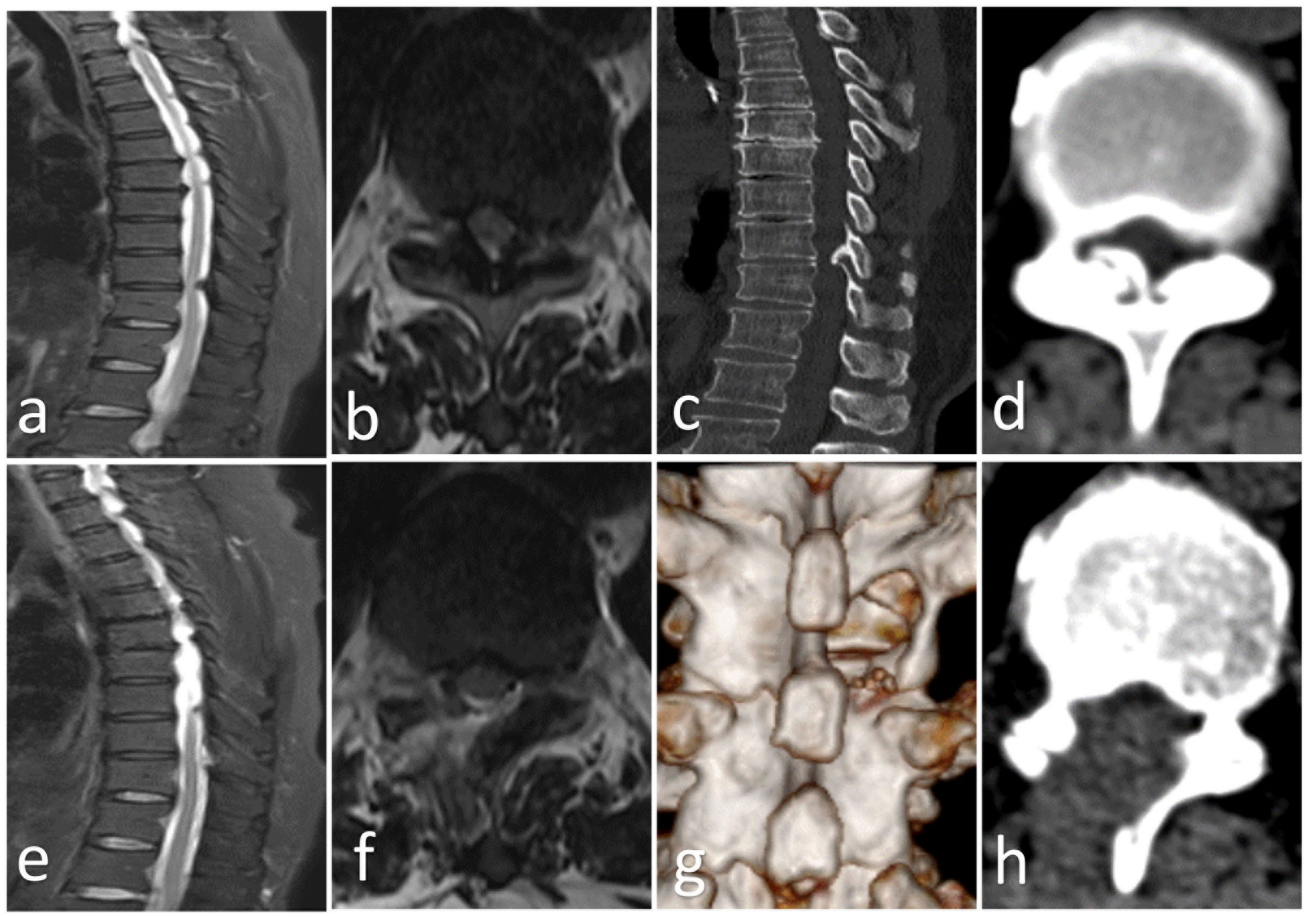
**(a,b)** Preoperative MRI shows ossification of the thoracic ligamentum flavum (Beak); **(c,d)** Preoperative CT shows ossification of the thoracic ligamentum flavum (Enlarged); **(e,f)** Postoperative CT shows that the ossification has been removed and spinal cord decompression has been successful; **(g,h)** Postoperative CT and 3D reconstruction show that the spinal canal has been opened and the ossification has been removed.

The patient is positioned prone, and anesthesia is administered using local anesthesia with intravenous adjuncts. Under fluoroscopic guidance with a C-arm, the target segment is localized and marked on the skin surface. After routine disinfection and draping with waterproof protection, an 18-gauge puncture needle was used to puncture at the marked point. The target point is located at the junction of the spinous process and the lamina. The incision was enlarged, the needle core was removed, and the guide wire was inserted. The dilator sheath was sequentially inserted, and the “U”-shaped beveled working sheath was finally placed. The position of the “U”-shaped sheath was confirmed under fluoroscopy using the C-arm. The fully visualized endoscope was inserted. First, a plasma radiofrequency electrode and nucleus pulposus forceps were used to expose the ipsilateral lamina and the base of the spinous process. Next, an eccentric ring was used to address the ipsilateral lamina and the base of the spinous process, and then the contralateral bone was removed through the roof, ranging from the head and tail to the starting and ending points of the ligamentum flavum, and extended bilaterally to the medial aspect of the superior articular processes. The deep lamina and ossification were subsequently thinned with a grinding drill, and the thinned ossification was removed with an Endo Kerrison rongeur. Finally, the bleeding was stopped fully, drainage was performed, the endoscope was removed, the wound was sutured, and the operation ended (Figure 3).

#### Evaluation index

Patient age, sex, surgical segment, hospitalization time, operative time and associated complications were recorded. Neurological status, both preoperatively and postoperatively, was evaluated using the mJOA score, the ASIA sense score (ASS), and the ASIA motor score of the lower extremities (AMS). The recovery rate (RR) was calculated as follows: RR = (postoperative JOA-preoperative JOA)/(11-preoperative JOA) × 100%. The surgical results were divided into good (50–100%), fair (25–49%), unchanged (0–24%), or deteriorated (<0%) according to the RR.

T-OLF was classified on the basis of sagittal MRI and axial CT. T-OLF can be classified as lateral, extended, enlarged, fused, or nodular on axial CT or as round or beak on the basis of sagittal MR images. After surgery, the degree of nerve decompression and articular process wear will be evaluated via 3D CT and MRI of the thoracic spine. All patients were followed up for at least 1 year.

### Statistical analysis

All the statistical analyses were performed using SPSS 22.0 software. The variables were statistically analyzed by the t test, and the chi-square test was used for categorical variables. A *p* value less than 0.05 was considered to indicate statistical significance.

## Results

### Basic information

All patients were followed up for an average of 12–24 months. 33 patients underwent UBE surgery, and 31 patients underwent PE surgery. There was no significant difference in baseline characteristics between the two groups (Table 1).

**Table 1:** Patient characteristics.

Characteristic	UBE (*n* = 33)	PE (*n* = 31)	*p* value
Age	60.12 ± 15.23	58.91 ± 14.79	0.58
Gender (Female/Male)	18/15	12/19	
BMI	26.18 ± 5.57	25.34 ± 6.31	0.91
Type (axial CT)			0.85
Lateral	3	1	
Extended	2	2	
Enlarged	10	9	
Fused	14	15	
Nodular	5	4	
Type (sagittal MRI)			0.48
Round	21	17	
Beak	12	14	
Operated level			0.52
Upper thoracic vertebrae (T1–T4)	6	5	
Middle thoracic vertebrae (T5–T9)	6	6	
Lower thoracic vertebrae (T10–T12)	19	20	
Operation time (min)	152.63 ± 50.98	198.79 ± 60.13	0.07
Hospitalization time	6.01 ± 1.01	5.12 ± 1.23	0.61

### Clinical efficacy

Neurological function was significantly improved in both groups after surgery. Compared with the PE group, the UBE group had better postoperative clinical efficacy, and the difference was statistically significant (*p* < 0.05). According to the RR, 24 patients were classified as good in the UBE group, 20 were classified as good in the PE group, and no patients deteriorated in either group (Table 2). Postoperative CT and MRI revealed that spinal cord compression in patients in both the UBE and PE groups was improved.

**Table 2:** Comparison of clinical efficacy between the two groups.

Characteristic	UBE (*n* = 33)	PE (*n* = 31)	*p* value
mJOA score			
Preoperative	6.12 ± 1.17	5.93 ± 1.21	0.27
Postoperative	7.53 ± 1.44^*^	6.98 ± 0.99^*^	0.01
Recovery Rate (RR)	40.12 ± 29.23^*^	39.87 ± 30.99^*^	0.03
Last Follow-up	8.79 ± 1.73^*^	8.41 ± 1.26^*^	0.21
ASS score			
Preoperative	175.23 ± 18.94	174.88 ± 19.72	0.54
Postoperative	202.97 ± 19.51^*^	200.68 ± 20.13^*^	0.03
Last Follow-up	203.88 ± 18.24^*^	201.33 ± 19.74^*^	0.59
AMS score			
Preoperative	35.18 ± 10.23	34.72 ± 11.44	0.13
Postoperative	46.57 ± 9.23^*^	44.18 ± 10.09^*^	0.00
Last Follow-up	47.19 ± 8.46^*^	45.73 ± 9.15^*^	0.68
**Outcome (by RR)**			0.59
Deteriorated	0	0	
Unchanged	3	5	
Fair	6	6	
Good	24	20	

^*^Compared with the preoperative value, *p* < 0.05.

### Complications

Four patients in the PE group and 1 patient in the UBE group had dural sac tears. Three patients with upper thoracic vertebrae in the PE group presented transient neurological deficits, including a temporary decrease in muscle strength, which resolved within 24 h. All patients were discharged after conservative treatment.

## Discussion

T-OLF of the spine progresses insidiously over a long period of time and can eventually cause myeloradiculopathy and pain (17). The standard surgical method for the treatment of T-OLF is resection of the posterior wall of the thoracic spinal canal (18). By removing the lamina of the corresponding segment and the medial half of the bilateral facet joints, along with the ossified ligamentum flavum, dorsal spinal cord compression can be fully relieved, and symptoms can be alleviated. The minimally invasive treatment of T-OLF is a feasible and promising surgical approach (19). Compared with traditional surgery, minimally invasive techniques operate within a continuous saline environment, utilizing the magnification provided by the endoscope to clearly identify anatomical structures (20). Additionally, these techniques offer advantages such as less trauma, reduced bleeding, minimized soft tissue damage, decreased impact on the spinal activity unit, and rapid recovery (21).

In our study, there was no significant difference in operation time or postoperative hospital stay between the two groups. However, in the PE group, local anesthesia was used, and the operator monitored the spinal nerve status through communication with conscious patients, which reduced the risk of iatrogenic nerve injury. In the UBE group, general anesthesia was used, requiring additional doctors to assess the spinal cord nerve status through neuroelectrophysiological monitoring during the operation, which increased medical expenses and, consequently, the economic burden on patients.

The mJOA scores, ASS scores, and AMS scores of the two groups in our study significantly improved compared with the preoperative values, both 3 days postsurgery and at the final follow-up. Therefore, we believe that both PE and UBE are effective in achieving spinal nerve decompression and relieving patients’ symptoms. Furthermore, compared with the PE group, the UBE group demonstrated superior postoperative clinical efficacy. This may be attributed to the fact that UBE avoids the limitations of the coaxial field of vision seen with PE by establishing two separate channels, thereby widening the field of view. The unfixed hard channel allows for greater and more flexible instrument movement, facilitating the removal of contralateral bone and ossified tissue.

Previous studies have indicated that dural sac tears are the most common complication in T-OLF surgery, and our findings corroborate these results (22, 23). However, the incidence of dural sac tears was greater in the PE group (4 patients) than in the UBE group (1 patient). This may be due to the uncertainties associated with using trephine and Endo-Kerrison instruments in the PE group, particularly when using the Endo-Kerrison to remove thin ossification under coaxial visualization. Accurately determining whether adhesion exists between the dura and the ossification can be challenging, which hinders the flexible removal of ossification. The UBE procedure effectively separates the working channel from the observation channel, enabling precise visualization, which reduces the incidence of dural tears. Therefore, we believe that UBE offers greater flexibility and precision in the removal of ossified material in patients with OLF accompanied by dural ossification. In cases of tight adhesion between the ossified material and the dural sac, it also allows complete “floating” of the spinal cord.

Although the clinical results of this study are satisfactory, the risk associated with surgical procedures in the upper thoracic vertebrae is greater. In our study, 6 patients in the UBE group had upper T-OLF, and none experienced postoperative neurological deficits. In the PE group, 5 patients had T-OLF in the upper thoracic vertebrae, and 3 of them experienced postoperative neurological deficits. The possible reason is that in PE, a circular saw is used to remove the ossified ligamentum flavum. The accuracy of circular saw resection is highly dependent on the surgeon’s experience, increasing the difficulty and uncertainty of the procedure and leading to a higher incidence of nerve neurostimulation. Therefore, we suggest that the upper thoracic vertebrae should preferentially be treated with UBE. Compared with the PE procedure, UBE offers greater stability. The use of a grinding drill in UBE for thoracic vertebral bone removal provides high controllability, a more precise decompression range, less spinal cord or nerve interference, and greater safety.

Our study has several limitations. First, patients with extralarge-type OLF and those with OLF at multiple vertebral levels were excluded, which may introduce publication bias regarding complications and operation time. Second, the sample size was small, and the follow-up period was relatively short. Multicenter, long-term follow-up studies are necessary to gather more robust clinical data in the future.

## Conclusion

For patients with T-OLF, both UBE and PE can effectively alleviate nerve compression and relieve symptoms. UBE uses two channels for observation and operation, which offers greater flexibility and allows for the continuous use of a grinding drill for bone decompression, leading to more effective and safer clinical outcomes.

## Data Availability

The raw data supporting the conclusions of this article will be made available by the authors, without undue reservation.
